# The Investigation of Radiographic Findings of Mandibles on Panoramic Radiographs of Patients with Primary Hyperparathyroidism Using Fractal Analysis

**DOI:** 10.5152/eurasianjmed.2021.20140

**Published:** 2021-10

**Authors:** Furkan Canturk, Ozkan Miloglu, Ismail Gumussoy, Ramazan Dayanan

**Affiliations:** 1 Department of Oral Dental and Maxillofacial Radiology, Atatürk University School of Dentistry, Erzurum, Turkey; 2 Department of Oral, Dental and Maxillofacial Radiology, Sakarya University School of Dentistry, Sakarya, Turkey; 3 Department of Endocrinology, Internal Medical Sciences, Atatürk University School of Medicine, Erzurum, Turkey

**Keywords:** Primary hyperparathyroidism, trabecular bone, fractals, panoramic radiography

## Abstract

**Objective:**

The basis of the research is the application of fractal analysis (FA) to panoramic radiographs of patients with primary hyperparathyroidism (PHPT). In addition, the relationship between the relevant biochemistry parameters and fractal values of healthy controls and patients with PHPT will be evaluated.

**Materials and Methods:**

In the current study, FA was performed with ImageJ program on panoramic radiographs of 48 patients diagnosed with PHPT. Fractal dimension (FD) values of the patients and 48 healthy subjects were compared. In addition, biochemical [parathyroid hormone (PTH), serum calcium (Ca), phosphorus (P), alkaline phosphatase (ALP), and vitamin D] parameters and FD values of both groups were analyzed with Mann–Whitney *U*-tests and Pearson correlation coefficient (*P* < .05).

**Results:**

FD values of four different angular areas were significantly lower in the patient group than in the healthy group (*P* < .05). There was no statistically significant difference between the patient and control groups in the measurements between the apexes of the right and left premolar and molar teeth. The mean PTH, Ca, P, and vitamin D values of the patients with PHPT and control group were highly significant, and all the parameters of the patient group showed higher values than the control group except for the *P* values (*P* < .001). In the patient group and control group, there was no significant difference between mean ALP values (*P* = .48). No correlation was found between the two groups.

**Conclusion:**

Bone biopsy is the gold standard method for the diagnosis of bone structure changes in patients with PHPT. However, it is not used routinely because it is an invasive method. The FA method applied to panoramic radiographs may be used as a noninvasive, easy-to-apply method to reveal the changes in the trabecular structure of the jaw bones of the patients.

## Introduction

Hyperparathyroidism is a disease that develops as a result of excessive parathyroid hormone (PTH) secretion of the parathyroid glands. Hyperparathyroidism has 3 forms: primary, secondary, and tertiary. Primary hyperparathyroidism (PHPT) is the 3rd most common endocrine disease and parathyroid gland adenoma or gland hyperplasia mostly play a role in disease etiology. There is a defect in the regulation of calcium (Ca) metabolism in PHPT. Recently, the use of biochemical tests such as PTH, serum Ca, phosphorus (P), and alkaline phosphatase (ALP) levels has become increasingly widespread in medicine, and as a natural result, the number of patients diagnosed without any signs or symptoms is increasing.^1,2^

The parathyroid glands, bones, and intestines interact with each other and play a role in the formation of mineral and bone disorders. So many changes occur in the bone structure due to hyperparathyroidism, stimulation of osteoclastic and osteoblastic activity, irregularly processed abnormal osteoid structure, fibrosis and cyst formation, decreased bone strength, bone pain and fractures. Osteoporosis, which develops as a secondary effect of PHPT, has some negative effects such as increase in demineralization and decrease in trabeculation in the mandibular bone as in other bones in the body.^3-^^6^

Cancellous bone is metabolically more active, and it has a higher bone-cycle rate than compact bone.^7^ Thus, it is better indicator of metabolic activity and it presents more valuable diagnostic information than compact bone. Also, cancellous bone presents fractal properties such as self-similarity, complexity, and characteristic length due to its natural architecture. Therefore, fractal analysis (FA) may be used to describe the complex structure of trabecular bone.^8,9^ The texture of images consists of many small components that present fractal features. Many methods have been developed for analysis of this texture by researchers. FA is an image analysis method used to describe complex shapes and structures.^8^ In recent years, the use FA has expanded in many scientific fields such as biology and medicine.^10^ It has been reported that FA of trabecular bone is a practical, low-cost, and promising tool for the evaluation of bone tissue in recent studies in the literature.^8,11–13^ Researchers used the FA method to evaluate jaw bones in various osteoporotic diseases, such as primer osteoporosis, chronic renal failure, systemic steroid use, or bisphosphonate intake. They stated that FA may distinguish patients with osteoporotic diseases from healthy controls.^8,14–16^

In this study, it was hypothesized that trabecular bone structure of mandible in patients with PHPT would decrease according to healthy individuals. With the aim of this, trabecular bone structure of mandible was examined on dental panoramic radiographs using fractal box-counting method. Although there are many studies on the evaluation of the osteoporotic effects of various metabolic and endocrine diseases on the jaw bones in the literature, there is only one case report related to PHPT. According to our knowledge, this paper was the first research study in the literature related to the FA method and evaluation of mandibular bone structure in patient with PHPT.

## Materials and Methods

### Study Group

Present study was performed using panoramic radiography of patients diagnosed as PHPT in Internal Medicine Service. Study was approved with decision number 30/2019 by the local ethics committee, and all participants completed the written informed consent. A number of patients with osteoporotic pathologies that could affect bone metabolism, such as primer osteoporosis, renal failure, diabetes mellitus, systemic steroid use, or bisphosphonate intake, were not included due to their medical condition. Forty-eight patients who were treated for hyperparathyroidism were included to study. The age range of patients was 23-71 years (mean age is 53 years). Seven patients were male and 41 patients were female. Control group consisted of 48 age- and sex-matched people with no systemic disease who underwent radiographic examination for various dental problems. The age range of controls was 29-73 years, with a mean age of 53 years. All procedures performed in this study were in accordance with the Helsinki Declaration of 1964 and later versions.

### Biochemistry

Biochemical parameters, including PTH, serum Ca, P, ALP, and vitamin D, were recorded within the last 3 months. Beckman Coulter AU-5800 and DXI-800 (Beckman Coulter Inc., Brea, CA) chemistry analyzers were used in the Biochemistry Laboratory of Ataturk University.

### Images and Fractal Analysis

All dental radiographic examinations were performed with using the same panoramic radiography device (ProMax^®^; Planmeca Oy, Helsinki, Finland) and the same X-ray technician. The exposure parameters were set on average as 62 kVp, 4 mA, and 16.2 s.

**Figure 1.**
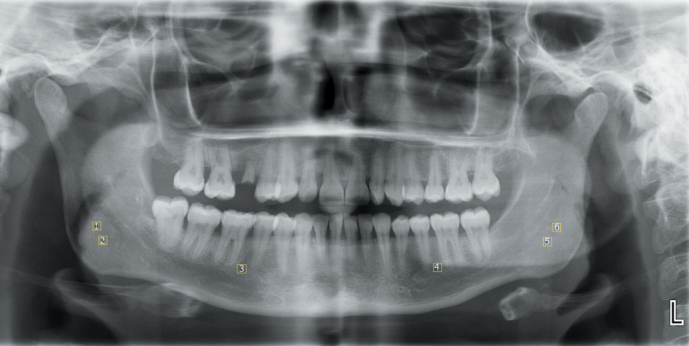
ROI selection on panoramic radiography to be used for FA. 385 × 194 mm (120 × 120 DPI)

**Figure 2.**
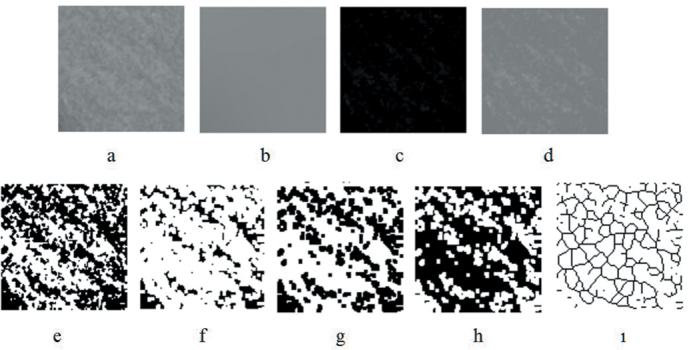
FD analysis transactions. (a) Duplicated ROI. (b) Blurred image duplicated ROI. (c) The blurred image was then subtracted from the original image. (d) Adding 128 to the result. (e) Application of 128 threshold value. (f) Erosion process. (g) Dilatation process. (h) Reversing. (ı) Skeletonizing 237 × 119 mm (120 × 120 DPI)

**Figure 3.**
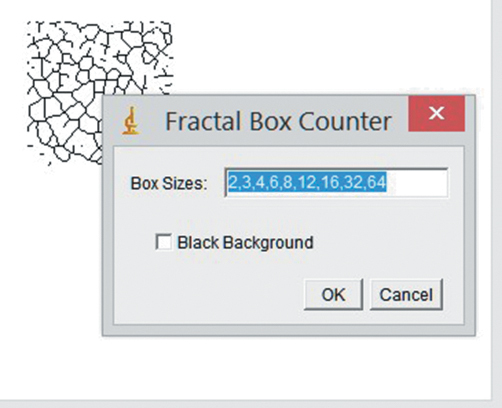
The last step of FD analysis transactions: skeletonizing is seen on the left side and calculation of fractal dimension is seen on the right side. 77 × 62 mm (120 × 120 DPI)

**Figure 4.**
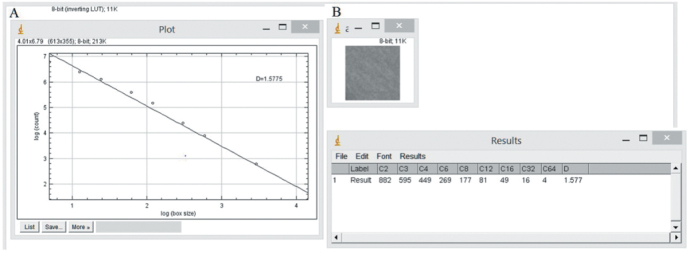
(A) Preparing the slope of the line giving the fractal dimension value. (B) Numerical representation of the fractal dimension value. 149 × 55 mm (220 × 220 DPI)

Box-counting method as designed by White and Rudolph^14^ was used to perform FA on the mandible. Six regions of interest (ROIs) were chosen on both sides of the mandible, and the size of every ROI was set to 35 × 35 pixels. ROI 1, 2, 5, and 6 were chosen on the mandibular angular region and, ROI 3 and 4 were chosen between the first molar and second premolar tooth apexes of the mandible (Figure 1). Boundary of compact bone and anatomical structures, such as tooth root, mandibular foramen, and mandibular canal, were not included to ROIs, only cancellous bone was selected. In addition, conditions such as periapical lesion and thickening of the lamina dura were excluded from the study. FA transactions (Figures 2-4) were performed by the same person and ImageJ 1.52b image analysis software (National Institutes of Health, Bethesda, MD; https://imagej.nih.gov/ij/index.html).

### Statistical Analysis

A single investigator [first author of this article (a 4-year experienced dental radiologist)] evaluated all parameters. To evaluate the intra-observer reliability, image analysis was repeated twice on 20 randomly selected radiographs by the same investigator, and intraviewer reliability was 95%.

Results were analyzed using SPSS statistic program version 22.0 (IBM SPSS Corp.; Armonk, NY, USA). The Mann–Whitney *U*-test was performed in the comparison of fractal dimension (FD) values of patient with PHPT and the control subjects. The Pearson correlation was performed in the analysis of the correlation between the biochemical parameters and FD values of patients. Results were reported as mean ± standard error, and a level of *P* < .05 was accepted as statistically significant.

## Results

According to outcomes of present study, FD values of the angular regions of patients with PHPT were lower than the healthy controls, and there were statistical significance in outcomes (*P* < .05). The mean FD values of patients were 1.2433, 1.2669, 1.28233, and 1.2804 for ROI 1, 2, 5, and 6, respectively. The mean FD values of controls were 1.2952, 1.3094, 1.3219, and 1.3031 for ROI 1, 2, 5, and 6, respectively. However, there was no correlation between FD values of premolar-molar regions of patients and controls (*P* = .846, *P* = .851). The mean FD values of patients were 1.2809 and 1.2744 for ROI 3 and 4, respectively. The mean FD values of controls were 1.2710 and 1.2950 for ROI 3 and 4, respectively (Table 1).

The mean PTH levels of the controls and patients were 44.02 ± 24.3 and 143.77 ± 47.9 pg/mL, respectively. The mean serum Ca levels of the controls and patients were 11.00 ± 0.6 and 9.23 ± 0.5 mg/dl, respectively. The mean serum P levels of the controls and patients were 3.73 ± 0.6 and 2.55 ± 0.4 mg/dl, respectively. The mean ALP levels of the controls and patients were 96.95 ± 31.2 and 109.27 ± 57.9 U/L, respectively. The mean vitamin D levels of the controls and patients were 27.47 ± 12.6 and 14.04 ± 6.7 ng/mL, respectively. The mean values of the biochemical parameters of the patients and control group (as the average of the last 3 months; PTH, Ca, P, ALP, and vitamin D) are given in Table 2.

In the comparison of FD values and biochemical parameters of patients, there was no significant correlation (Table 3). In addition, there was no correlation between FD value, gender and age.

## Discussion

In the literature, studies with morphometric measurements of mandibular bone have mostly evaluated osteoporosis. Also in dentistry, radio-morphometric indices were used to evaluate quality and quantity of bone particularly in implant cases. Current study is the first study involving FA performed on panoramic radiographs of patients with PHPT. According to the results, FD values of four different angular areas were significantly lower in the patient group than in the healthy group. There was no statistically significant difference between the patient and control groups in the measurements between the apexes of the right and left premolar and molar teeth. One of the reasons may be that different parts of the bone are affected differently from the disease.

The measurements of bone quantity are simpler than assessing bone quality. The term of bone quality includes so many determinant such as spatial bone geometry, microarchitecture of bone tissue, trabecular continuity, bone mineral deposit, and bone mineral defects. Most of the changes caused by all these factors on the bone structure are not visible on the radiography with the naked eye. Bone mineral density measurement using with dual-energy X-ray absorptiometry had been used frequently in the analysis of bone structure in the past. With the widespread use of computed tomography, HU measurement has become popular as a more practical and preferable method in the measurement of bone mineral density.^17^ On the other hand, current studies have shown that the measurement of bone mineral density alone is not sufficient for the evaluation of bone quality and bone microstructure analysis should be considered.^18,19^

FA; conversion of formal similarities in organic or inorganic structures into numerical values by means of morphometry and mathematical connections. Microstructure of bone presents fractal properties, and it can be evaluated using the FA method.^10^ The application field of FA is very wide from mathematics to biology and has been also used in numerous studies in dentistry. The FA method has been used in previous studies for the analysis of trabecular microstructure in periodontal disease, osteoporosis, and implant planning.^20–23^ In a study-related periodontitis and alveolar bone loss, Updike and Nowzari^8^ reported that the destructive effects of periodontitis on trabecular bone may be successfully evaluated with FA method. Jett et al.^24^ stated that even small destructive changes on trabecular bone in the early stages of pathology may be determined by the FA method before the effects of periodontal destruction are emerged. However, Sahin et al.^25^ detected no significant difference the FD values between early-stage and advanced-stage medication-related osteonecrosis of the jaw. The differences between the FD values reported by Sahin et al.^25^ and Jett et al.^24^ may have been influenced by the discrepancies in the selection of ROIs, sizes of ROIs, and patient differences. Therefore, more follow-up studies performing FD analysis on the radiographs of patients are needed.

PHPT is a disease characterized by increased serum Ca and PTH levels. Osteoclastic activation and destruction already present in the bones are increased in hyperparathyroidism. Many clinical studies and reviews have reported that hyperparathyroidism negatively affects skeletal BMD. Metabolic bone diseases such as hyperparathyroidism can affect the entire skeletal system, as well as the maxilla and mandible, and increase the severity of periodontal diseases.^26^ In a study conducted with cone beam computed tomography (CBCT) images of patients exposed to secondary hyperparathyroidism due to chronic renal failure, it was stated that osteoporotic bone changes were more prominent in the jaw bones of these patients.^27^

Outcomes of this study are partly consistent with previous studies conducted with the patients with osteoporotic pathologies. Gumussoy et al.,^16^ in a study conducted with panoramic radiographs of patients with renal failure, stated that the FD values of the patients with renal failure were lower compared the healthy individuals. Researchers associated their result with decrease of complexity in bone microstructure and they stated that the osteoporotic effect of renal failure leads to a more smooth form in trabecular microstructure. In another study conducted with panoramic radiographs of patients taking bisphosphonates, Demiralp et al.^28^ stated that the FD values of the patients were higher than FD values of the healthy individuals. The outcomes of this study can be related with the antiresorptive effects of bisphosphonates on the bone structure. Updike and Nowzari^8^ also reported that the destruction process started with periodontal pathologies cause a decrease in complexity of bone microstructure and FD value.

Previous studies related to FA in the literature have reported that a higher FD value indicates a more complex structure in trabecular bone architecture.^29^ However, different FA outcomes have been obtained in many different studies. Because, there is a lack of consensus on how pathologic bone change caused by diseases affects trabecular complexity and how it affects outcome of FA. While some of researchers (Hua et al.^30^ and Ruttimann et al.^31^) have stated that FD values increase in diseases that cause osteoporosis-like effect on bone structure, some of researchers (Southard et al.,^13^ Updike and Nowzari,^8^ Demirbaş et al.,^32^ and Ergün et al.^33^) have come to conclusion that FD values decrease. Our argument is that dilution in trabeculaes due to osteoporosis in PHPT, decrease in the number of trabeculaes, and increase the dimension of lacunaes between trabeculaes will decrease the complexity of the trabecular microstructure and FD values. However, changes such as thickening and resorption in trabeculaes affecting the FD values depend on many factors in bone turnover. Also this matters such as course of obtaining the images, criteria’s while determining the ROI, anatomical variations, how disease affects bone structure, how the jaw bones are affected by diseases in different parts of the body, and different methods used to obtain FD values can contribute the contradictory of the outcomes of FA studies.

Although there are many studies on the evaluation of the osteoporotic effects of various metabolic and endocrine diseases on the jaw bones in the literature, there is only one case report related to PHPT.^33^ In this study, FD analysis within a 5-year period was applied to the panoramic films of a patient with hyperparathyroidism, and his bone structure was evaluated in a non-invasive fashion. They have reported that after the removal of a parathyroid adenoma; mandibular bone FD analyses revealed a prominent development. On the other hand, FD increased significantly by the 2-year follow-up examination, showing the effect of the operation on the mandibular bone. In conclusion, the results confirmed in this research that FD analysis is a practical method to investigate trabecular bone architecture and FD values decrease due to osteoporosis in PHPT.

The use of panoramic radiography was one of the limitations of this study when compared to CBCT system. CBCT is a reliable diagnostic tool that does not involve many negative factors when compared with panoramic radiographs for assessment of bone structure. However, CBCT was not used in this study because it is not a routine radiographic method in dentistry, and CBCT emits much more radiation compared with panoramic radiography. On the other hand, panoramic radiography is the most preferred, simple, practical, and inexpensive imaging method for patients who consult a dentist for any reason.^34^ Another limitation of this study was that lack of histopathological assessment of how osteoporosis-induced changes in bone tissue affects trabecular structure in patients with hyperparathyroidism. The practice of bone biopsy is the gold standard on the evaluation of trabecular structure in metabolic bone diseases.^35^ However, bone biopsy is a non-practical, invasive, painful, and expensive method. In routine procedure, biochemical markers are used in medicine on the diagnosis, treatment, and follow-up of hyperparathyroidism. Therefore, the mean PTH, serum Ca, P, ALP, and vitamin D levels of patients with PHPT were used in present study to compare with FD values. There was not statistically significance between the FD values and biochemical markers related with PHPT.

In conclusion, current study performed the FA of microstructure of mandibular trabecular bone in patients with PHPT and compared with control individuals. FD values were found to be lower in angular region of mandible in patients with PHPT compared to healthy controls. There was not statistically significance on FD values of ROIs obtained from dentulous region. Researchers should consider that the trabecular bone structure may vary depending on occlusal stress in dentulous regions. The outcomes of present study suggest that the FA method may be a simple and practical tool to revealing the microstructural changes in trabecular bone. Further studies with histopathological findings should be performed as the gold standard to demonstrate the clinical validity of the FA method.

**Main Points**

- The aim of this study was to evaluate the mandibular trabecular bone of patients with PHPT using FA by comparing with healthy controls.
- FD values were found to be lower in angular region of mandible in patients with PHPT compared to healthy controls. Researchers should consider that the trabecular bone structure may vary depending on occlusal stress in dentulous regions.
- Bone biopsy is the gold standard method for the diagnosis of bone structure changes in patients with PHPT. However, it is not used routinely because it is an invasive method. Panoramic radiography is the most widely used imaging modality in dentistry and contains many clinical data related bone structure of patients. By using the FA method, data capacity of panoramic radiography may be utilized more in the evaluation of bone structure.

## Figures and Tables

**Table 1.** Mean FD Values of the Patients with PHPT and Healthy Individuals and the Mann−Whitney *U*-Tests Results

		ROI 1	ROI 2	ROI 3	ROI 4	ROI 5	ROI 6
Healthy controls	Mean	1.295277	1.309483	1.271085	1.295037	1.321969	1.303144
	SD	0.0768177	0.0555282	0.0592290	0.0691698	0.0558428	0.0879765
Patients with PHPT	Mean	1.243358	1.266927	1.280913	1.274481	1.283313	1.280463
	SD	0.0918985	0.0894960	0.0618507	0.0718596	0.0795098	0.0778094
*P* < .05		.011	.048	.373	.105	.026	.007

**Table 2.** Pearson Correlations and the Mean Values of Biochemical Parameters of the Patients with PHPT and Healthy Individuals

		Parathyroid hormone	Calcium	Phosphorus	Alkaline phosphatase	Vitamin D
Healthy controls	Mean	44.0250	9.2333	3.7396	96.9583	14.0431
	SD	24.34545	0.51004	0.66994	31.23484	6.79697
Patients with PHPT	Mean	143.7771	11.0000	2.5542	109.2708	27.4733
	SD	47.94983	0.67288	0.43121	57.92943	12.69566
*P* < .001		.000	.000	.000	.489	.000

**Table 3.** Pearson Correlations of FD Values of the Patients with PHPT and Biochemical Parameters

		ROI 1	ROI 2	ROI 3	ROI 4	ROI 5	ROI 6	Ca	P	Vitamin D	PTH
ROI 1	Pearson correlation	1	0.494^**^	0.130	0.232	0.031	−0.119	0.112	0.080	−0.076	0.015
	Sig. (2-tailed)		0.000	0.380	0.112	0.837	0.421	0.450	0.590	0.607	0.919
	N	48	48	48	48	48	48	48	48	48	48
ROI 2	Pearson correlation	0.494^**^	1	0.027	−0.069	−0.011	−0.220	−0.004	−0.152	−0.124	−0.002
	Sig. (2-tailed)	0.000		0.858	0.640	0.941	0.133	0.976	0.301	0.402	0.989
	N	48	48	48	48	48	48	48	48	48	48
ROI 3	Pearson correlation	0.130	0.027	1	0.142	0.247	−0.058	−0.064	0.181	0.186	0.066
	Sig. (2-tailed)	0.380	0.858		0.334	0.090	0.695	0.667	0.218	0.206	0.657
	N	48	48	48	48	48	48	48	48	48	48
ROI 4	Pearson correlation	0.232	−0.069	0.142	1	−0.031	−0.101	−0.080	0.323^*^	0.028	0.291^*^
	Sig. (2-tailed)	0.112	0.640	0.334		0.834	0.496	0.589	0.025	0.849	0.045
	N	48	48	48	48	48	48	48	48	48	48
ROI 5	Pearson correlation	0.031	−0.011	0.247	−0.031	1	0.275	0.017	−0.062	0.305^*^	−0.132
	Sig. (2-tailed)	0.837	0.941	0.090	0.834		0.058	0.908	0.675	0.035	0.373
	N	48	48	48	48	48	48	48	48	48	48
ROI 6	Pearson correlation	−0.119	−0.220	−0.058	−0.101	0.275	1	−0.069	−0.232	0.210	−0.182
	Sig. (2-tailed)	0.421	0.133	0.695	0.496	0.058		0.640	0.113	0.152	0.215
	N	48	48	48	48	48	48	48	48	48	48
Ca	Pearson correlation	0.112	−0.004	−0.064	−0.080	0.017	−0.069	1	0.116	0.086	−0.122
	Sig. (2-tailed)	0.450	0.976	0.667	0.589	0.908	0.640		0.434	0.562	0.409
	N	48	48	48	48	48	48	48	48	48	48
P	Pearson correlation	0.080	−0.152	0.181	0.323^*^	−0.062	−0.232	0.116	1	−0.126	0.517^**^
	Sig. (2-tailed)	0.590	0.301	0.218	0.025	0.675	0.113	0.434		0.395	0.000
	N	48	48	48	48	48	48	48	48	48	48
Vitamin D	Pearson correlation	−0.076	−0.124	0.186	0.028	0.305^*^	0.210	0.086	−0.126	1	−0.217
	Sig. (2-tailed)	0.607	0.402	0.206	0.849	0.035	0.152	0.562	0.395		0.139
	N	48	48	48	48	48	48	48	48	48	48
PTH	Pearson correlation	0.015	−0.002	0.066	0.291^*^	−0.132	−0.182	−0.122	0.517^**^	−0.217	1
	Sig. (2-tailed)	0.919	0.989	0.657	0.045	0.373	0.215	0.409	0.000	0.139	
	N	48	48	48	48	48	48	48	48	48	48

* Correlation is significant at the 0.05 level (2-tailed).

** Correlation is significant at the 0.01 level (2-tailed).

Ca: calcium, P: phosphorus, PC: Pearson correlation, PTH: parathyroid hormone, Sig.: significance
